# Calcium homeostasis and endoplasmic reticulum stress are involved in Salvianolic acid B-offered protection against cardiac toxicity of arsenic trioxide

**DOI:** 10.18632/oncotarget.22127

**Published:** 2017-10-26

**Authors:** Jing-Yi Zhang, Bin Zhang, Min Wang, Wei Wang, Ping Liao, Gui-Bo Sun, Xiao-Bo Sun

**Affiliations:** ^1^ Institute of Medicinal Plant Development, Peking Union Medical College and Chinese Academy of Medical Sciences, Beijing, China; ^2^ Beijing Key Laboratory of Innovative Drug Discovery of Traditional Chinese Medicine (Natural Medicine) and Translational Medicine, Beijing, China; ^3^ Key Laboratory of Bioactive Substances and Resource Utilization of Chinese Herbal Medicine, Ministry of Education, Beijing, China; ^4^ Zhongguancun Open Laboratory of The Research and Development of Natural Medicine and Health Products, Beijing, China; ^5^ College of Pharmacy, Guilin Medical University, Guilin, China

**Keywords:** arsenic trioxide, cardiotoxicity, salvianolic acid B, calcium homeostasis, endoplasmic reticulum stress

## Abstract

Arsenic trioxide (ATO) is a potent anticancer agent used to treat acute promyelocytic leukemia. However, its cardiotoxicity limits ATO’s widespread clinical use. Previous studies demonstrated that ATO may aggravate Ca^2+^ overload and promote endoplasmic reticulum stress (ERS). Salvianolic acid B (Sal B) is cardioprotective against ATO and enhances ATO’s anticancer activities. The present study assessed whether the Sal B protective effect was related to maintenance of Ca^2+^ homeostasis and inhibition of ER stress. Male BALB/c mice were injected with ATO or ATO+Sal B once a day via the tail vein for 2 weeks. We then detected the effects of Sal B in real time using adult rat ventricular cardiomyocytes *in vitro* using an IonOptix MyoCam system. Sal B treatment alleviated ATO-induced abnormal cardiac contractions and Ca^2+^ homeostasis imbalance. Sal B increased sarcoplasmic reticulum Ca^2+^-ATPase (SERCA) activity, regulated Ca^2+^ handling protein expression, and decreased expression of ERS proteins. Our results demonstrate that the cardioprotective effect of Sal B correlates with SERCA modulation, maintenance of Ca^2+^ homeostasis, and inhibition of ER stress. These findings suggest Sal B may ameliorate ATO cardiotoxicity during clinical application.

## INTRODUCTION

Arsenic trioxide (ATO) was recently found to induce complete remission in relapsed or refractory acute promyelocytic leukemia (APL) patients, and the US Food and Drug Administration subsequently approved ATO as the frontline agent for APL treatment [1-3]. However, numerous clinical trials reported that chronic treatment with a therapeutic ATO concentration could cause severe cardiac arrhythmia, such as QT interval prolongation, T-U wave alternans, ST-T change, and torsades de pointes, or even sudden death [4-7]. This issue has limited the clinical usefulness of the drug. QT interval prolongation is the most common adverse effect, and is caused by delayed membrane repolarization in cardiomyocytes. Calcium current is the primary inward current in cellular membrane repolarization, and Ca^2+^ cycling regulates cardiac contraction. Increased [Ca^2+^]_I_ is also a critical sign of cell apoptosis, and has been associated with a number of abnormalities in cardiac tissues, such as ventricular arrhythmias and contractile dysfunction [8]. ATO can augment I_Ca, L_ density, triggering intracellular calcium overload and disorder, and ultimately inducing arrhythmia and cell apoptosis [9-11].

The endoplasmic reticulum (ER) is the major intracellular Ca^2+^ reservoir. Ca^2+^-binding chaperones mediate proper folding of proteins in the ER lumen [12]. Previous studies showed that ATO is an ER stressor, and ATO-induced cardiac cell death is associated with ER stress-mediated apoptosis [13-15]. We recently demonstrated that ATO destroyed adult rat ventricular myocyte (ARVM) contractile functions and intracellular calcium homeostasis, inducing ER stress-mediated apoptosis [16]. Therefore, regulating calcium homeostasis imbalance and inhibiting ER stress may reduce ATO-induced myocardial injury.

**Figure 1:**
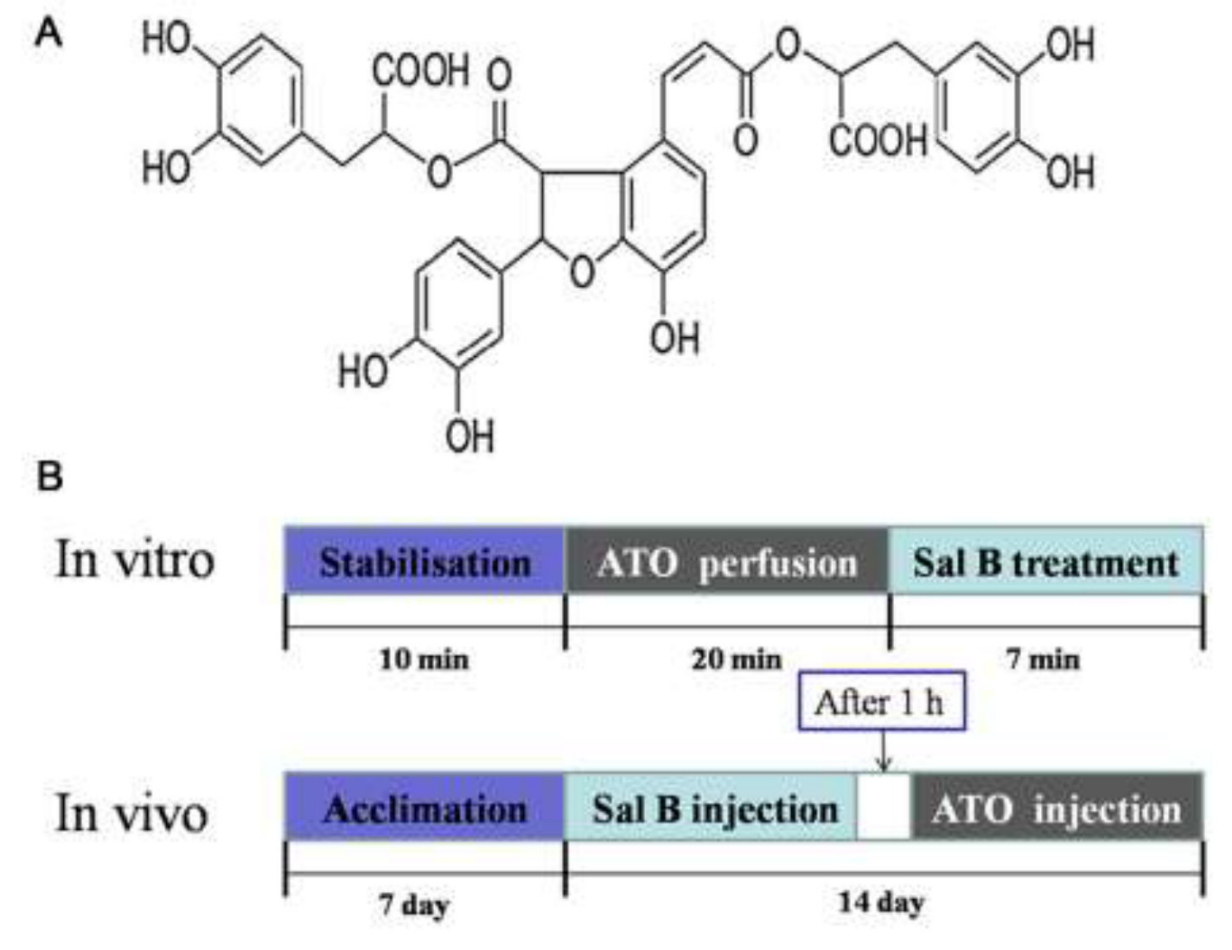
Sal B molecular structure **(A)** and *in vivo* and *in vitro* experimental designs **(B)**.

The dried root of *Salvia miltiorrhiza* Bunge (also known as Danshen) is a popular traditional Chinese medicine, and has been used extensively in both Asian and Western countries to treat various diseases, including cerebrovascular diseases, coronary artery diseases, and myocardial infarction [17]. Salvianolic acid B (Sal B; Figure 1) is the most abundant and bioactive compound in Danshen, and appears to have anti-oxidant, anti-apoptosis, and anti-tumor activities [18]. Our previous studies showed that Sal B attenuates ATO-induced cardiac injury via PI3K/Akt signaling in H9c2 cells [19]. Sal B also alleviates ATO-induced loss of cardiac function and damage to cardiomyocytic structures in BALB/c mice, and can improve ATO-induced cytotoxicity and apoptosis in HepG2 and HeLa cells [20]. These data suggest that Sal B and ATO act synergistically, with reduced cardiovascular damage.

Previous studies confirmed that Sal B inhibits Ca^2+^ channels and Ca^2+^ influx in rat coronary artery vascular smooth muscle cells [21]. Sal B reduces cytoplasmic calcium by modulating intracellular calcium release and extracellular calcium influx [22]. We hypothesized that the Sal B cardioprotective effects may also be associated with calcium overload inhibition and subsequent ER stress-mediated apoptosis suppression. This study assessed the effects of Sal B on cardiac contraction and Ca^2+^ transients in ARVMs in real time using an IonOptix MyoCam system. We repeated previous BALB/c mouse model experiments to elucidate the Sal B cardioprotective mechanism. We then assessed sarcoplasmic reticulum Ca^2+^-ATPase (SERCA) activity, and Ca^2+^-handling- and ER stress signaling-related proteins in heart tissue and ARVMs. Thus, we assessed whether Sal B protects against ATO-induced cardiotoxicity via calcium homeostasis maintenance and ER stress inhibition.

## RESULTS

### Effect of Sal B on cardiomyocyte contractile function after ATO treatment

**Figure 2:**
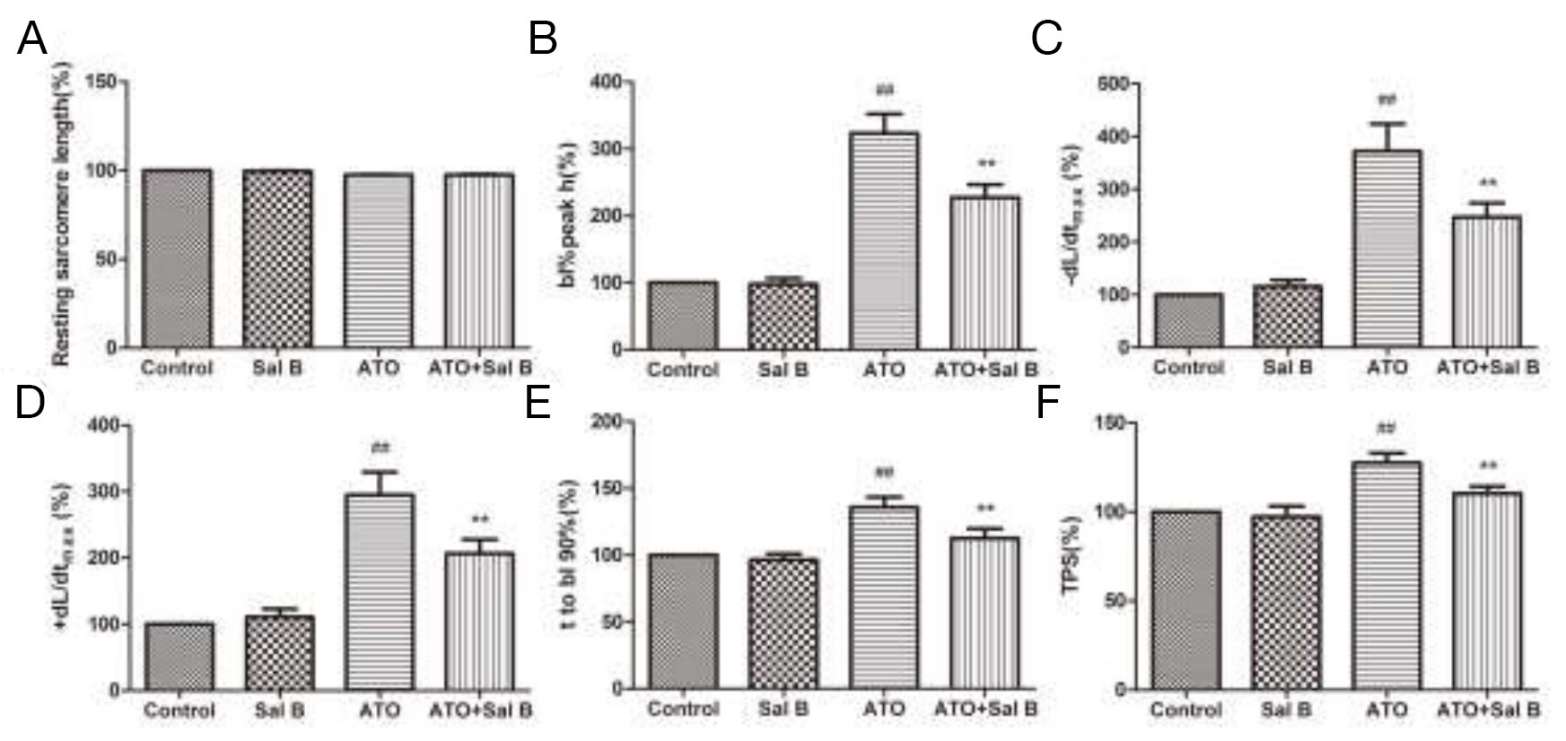
Effect of Sal B on cardiomyocyte contractile function after ATO treatment Resting sarcomere length **(A)** Sarcomere shortening amplitude **(B)** Relengthening maximal velocity (-dL/dtmax) **(C)** Shortening maximal velocity (+dL/dtmax) **(D)** Time-to-90% relengthening (TR90) **(E)** Time-to-peak shortening (TPS) **(F)** *n*=30–40 cells from 3 rats per group; ^##^p<0.01 vs. control, ^**^p<0.01 vs. ATO.

To determine whether Sal B protects sarcomeric contractile function against ATO-induced injury, ARVMs were treated with Sal B (1 μM) for 7 min after ATO treatment (100 μM) for 20 min. Sal B treatment alone did not affect resting cardiomyocyte contractile function compared with controls (Figure 2). However, Sal B and ATO-treated cells displayed normal sarcomere shortening amplitudes (Figure 2B), -dL/dt /+dL/dt (Figure 2C-2D), TR_90_ (Figure 2E), and TPS (Figure 2F), while ATO-treated cells (without Sal B) showed increased amplitudes. These data indicate that ATO perfusion severely impaired cardiomyocyte contractile function, and Sal B treatment eliminated this impairment.

### Effect of Sal B on intracellular Ca^2+^ transients after ATO treatment

**Figure 3:**
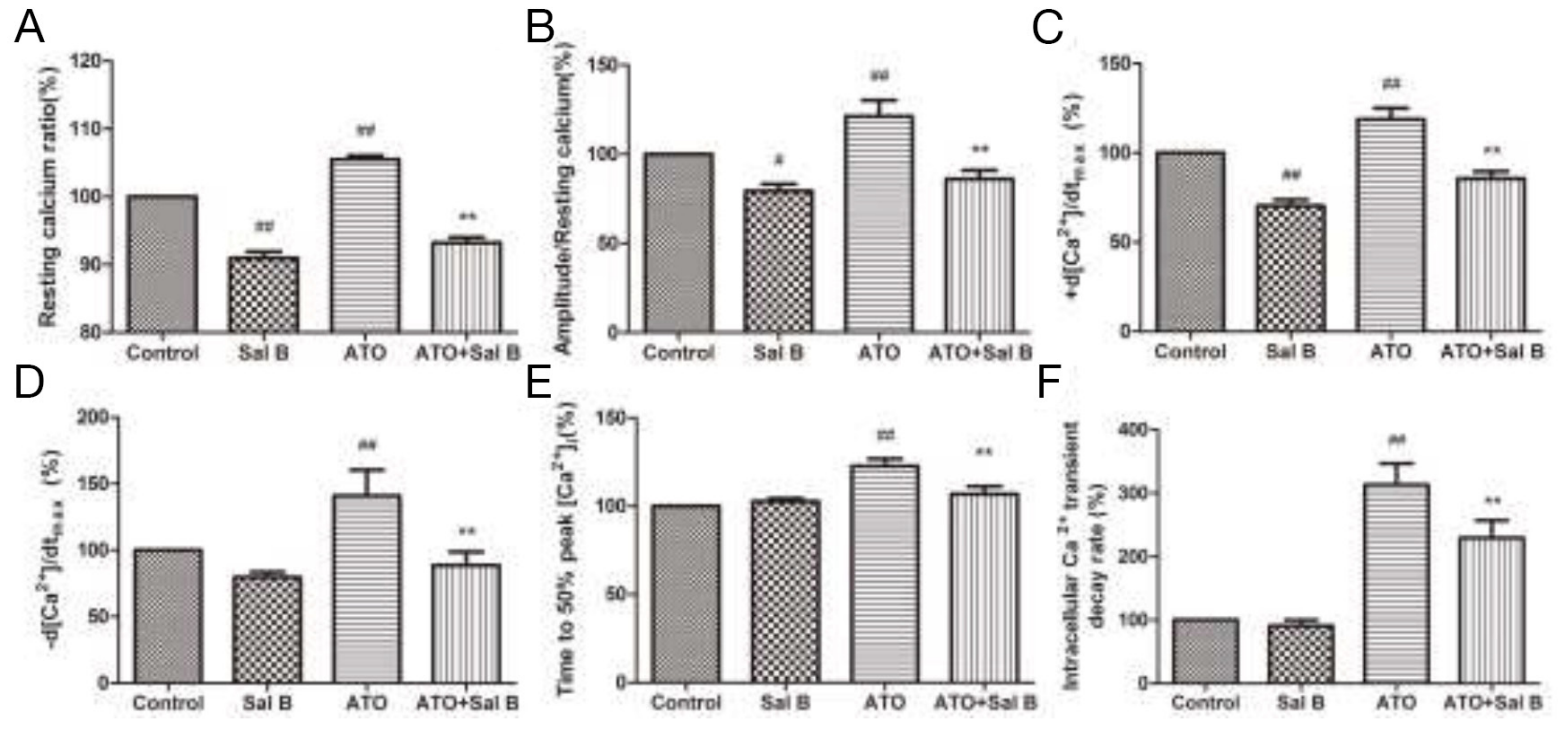
Effect of Sal B on ARVM intracellular Ca^2+^ transients after ATO treatment Resting calcium ratio **(A)** Amplitude/resting calcium **(B)** Ca^2+^ shortening maximal velocity (+d [Ca^2+^]/dtmax) **(C)** Ca^2+^ relaxation maximal velocity (-d [Ca^2+^]/dtmax) **(D)** Time-to-50% peak [Ca^2+^]_i_ **(E)** Intracellular Ca^2+^ transient decay rate **(F)** *n*=30–40 cells from 3 rats per group; ^#^p<0.05 vs. control, ^##^p<0.01 vs. control, ^**^p<0.01 vs. ATO.

We used intracellular fura-2 fluorescence to detect Ca^2+^ transients. As a calcium channel blocker [23], Sal B alone decreased the resting Ca^2+^ ratio and the amplitude of Ca^2+^ transients (Figure 3). Similar to previous findings [16], ATO treatment increased the resting Ca^2+^ ratio, amplitude, ±d [Ca^2+^]/dt_max_, time-to-50% peak [Ca^2+^]_i_, and [Ca^2+^]_i_ transients decay rate; Sal B treatment decreased those indexes (Figure 3A-3F). Sal B relieved Ca^2+^ overload and the intracellular calcium homeostasis imbalance induced by ATO in ARVMs.

### Effect of Sal B on SERCA activity after ATO treatment

**Figure 4:**
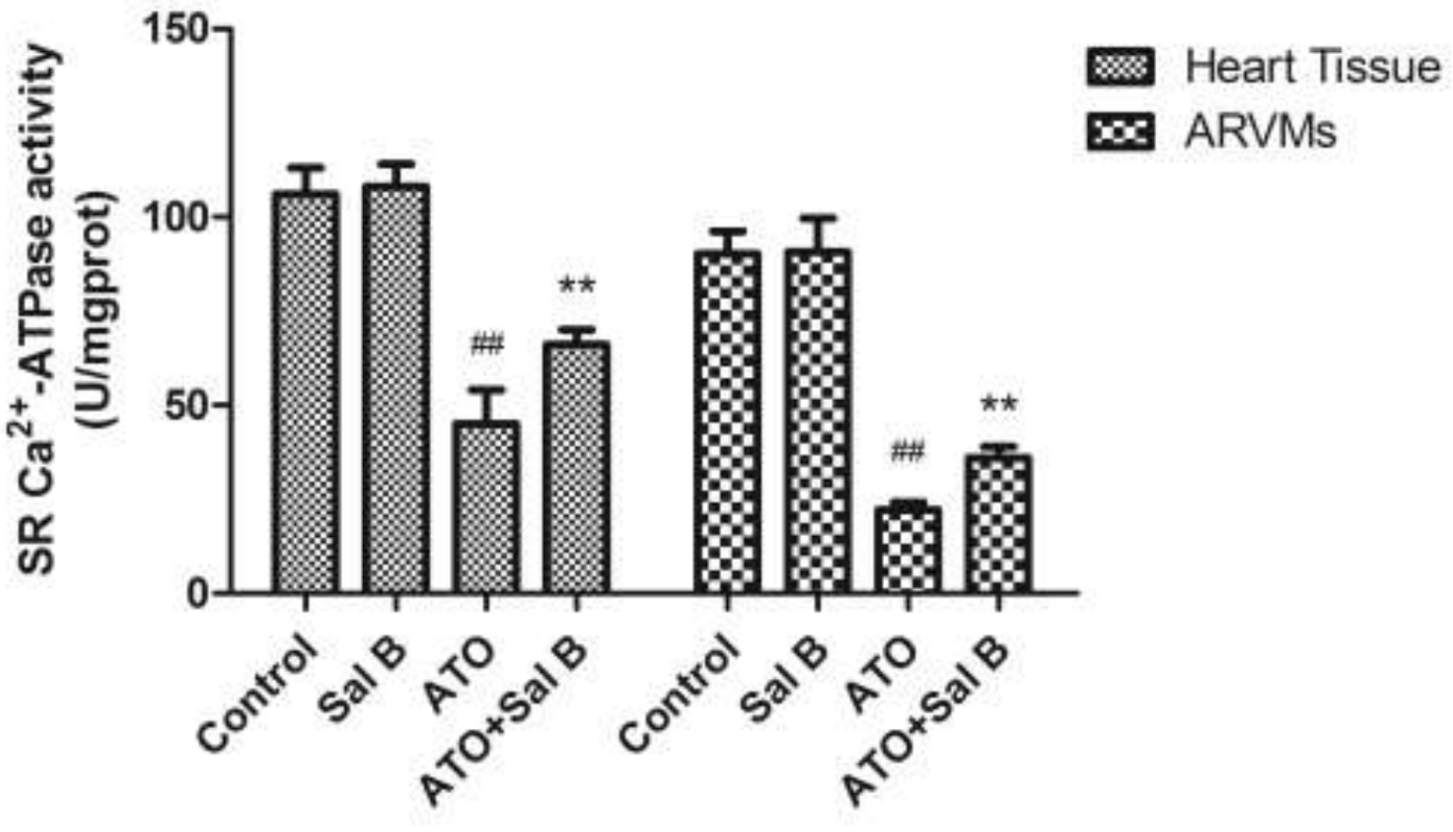
Effect of Sal B on SERCA activity after ATO treatment in heart tissue and ARVM Experiments were performed three times. ^##^p<0.01 vs. control, ^**^p<0.01 vs. ATO.

SERCA regulates cardiac muscle Ca^2+^ homeostasis and contractility. To determine whether Sal B cardioprotection involves SERCA, we examined SERCA activity in sarcoplasmic reticulum (SR) vesicles extracted from heart tissue and ARVMs. ATO attenuated SERCA activity, whereas Sal B treatment effectively reversed SERCA activity compared with controls (Figure 4). These results indicate that Sal B protects against ATO-induced injury by enhancing SERCA activity.

### Effect of Sal B on Ca^2+^ handling protein expression after ATO treatment

**Figure 5:**
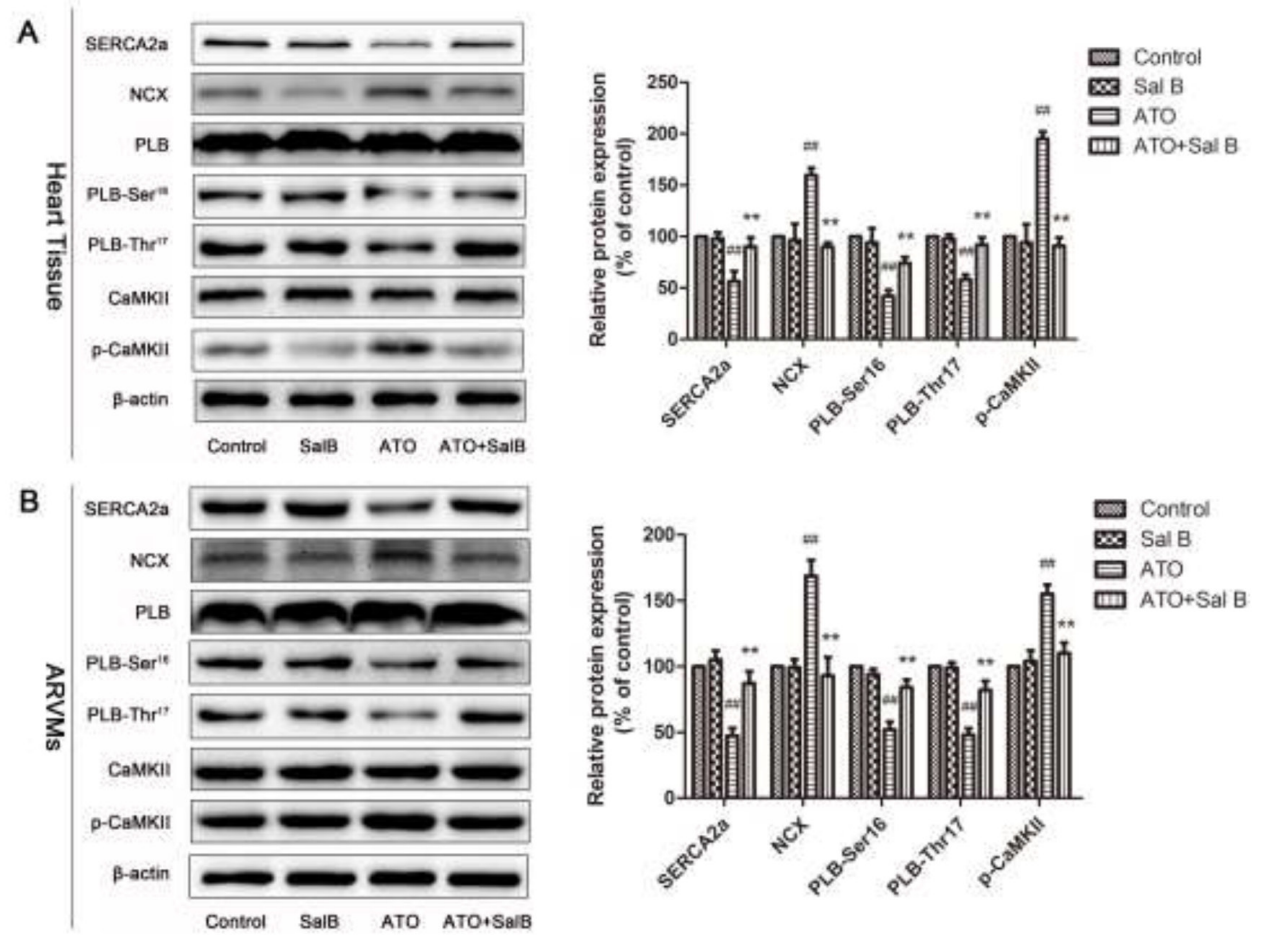
Effect of Sal B on Ca^2+^ handling protein levels after ATO treatment Ca^2+^ handling protein levels in heart tissue **(A)** and ARVMs **(B)** Experiments were performed three times. ^##^p<0.01 vs. control, ^**^p<0.01 vs. ATO.

We investigated Sal B regulation of Ca^2+^ handling proteins in heart tissue and ARVMs using western blotting. Consistent with our previous study [16], ATO treatment decreased SERCA2a, PLB, and phosphorylated PLB (p-PLB) levels and increased NCX and p-CaMKII levels (Figure 5). Sal B treatment blocked these changes, suggesting that Sal B regulates Ca^2+^-handling protein expression to protect against ATO-induced intracellular calcium homeostasis imbalance.

### Effect of Sal B on ER stress-related protein levels after ATO treatment

**Figure 6:**
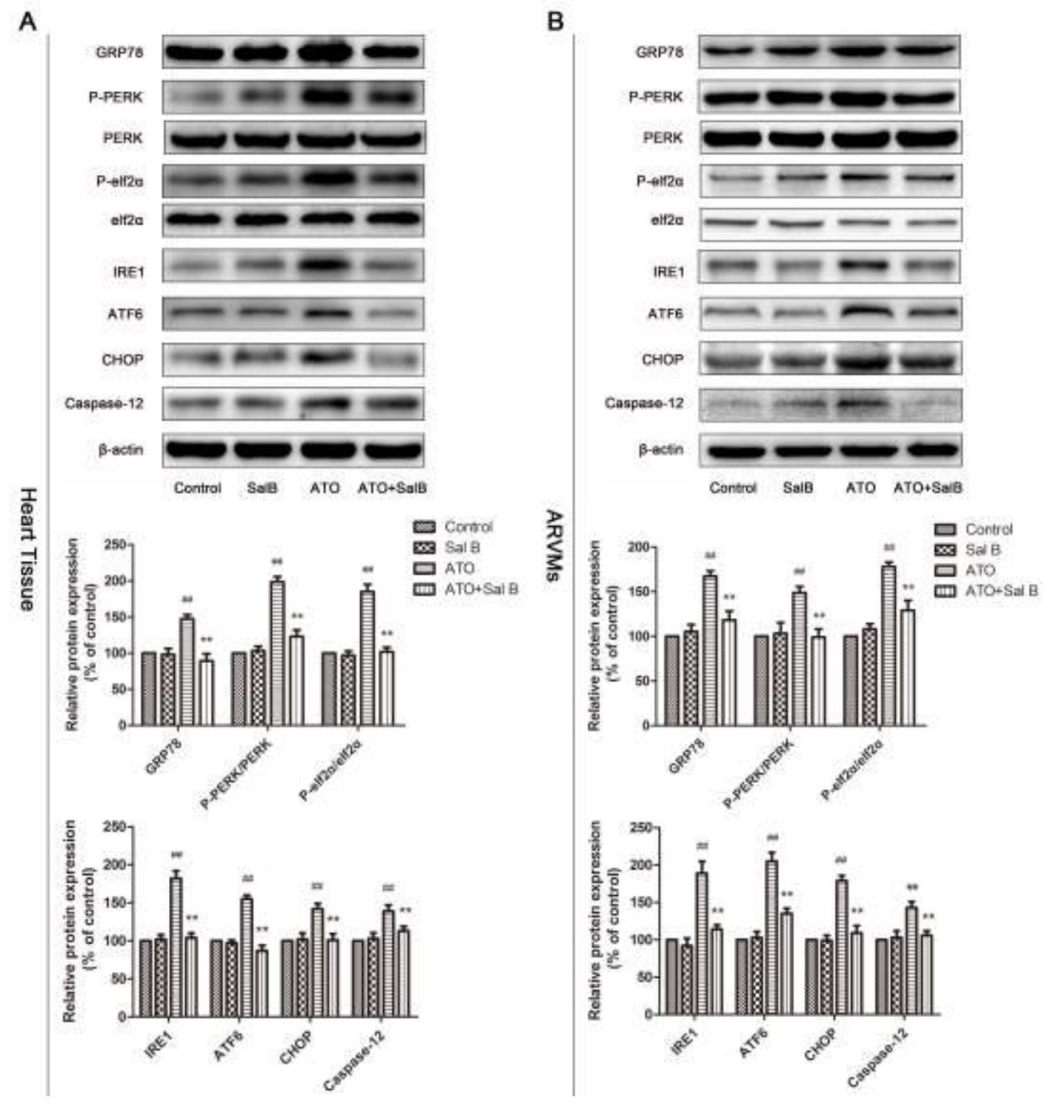
Effect of Sal B on ER stress-related protein levels after ATO treatment Ca^2+^ handling protein levels in heart tissue **(A)** and ARVMs **(B)** Experiments were performed three times. ^##^p<0.01 vs. control, ^**^p<0.01 vs. ATO.

To confirm the involvement of ER stress in Sal B cardioprotection, we evaluated an ER stress-responsive marker (GRP78), ER stress sensors (protein kinase RNA-like ER kinase (PERK), activating transcription factor 6 (ATF6), inositol-requiring enzyme-1α (IRE1), and eukaryotic initiation factor 2 α (eIf2α), and ER stress-initiated proapoptotic factors (C/EBP homologous protein (CHOP) and Caspase12) in heart tissue and ARVMs via western blotting. GRP78, p-PERK, p-eIf2α, IRE1, and ATF6 were upregulated in the ATO group compared with controls (Figure 6). However, Sal B treatment reduced expression of GRP78 and ER stress sensors compared with the ATO group, and suppressed ATO-induced CHOP and caspase-12 upregulation. Together, these results show that ER stress was associated with ATO-induced cardiotoxicity via CHOP and caspase-12 activation through three ER stress sensors: ATF6, PERK, and IRE1. Sal B suppressed ATO-induced ER stress and apoptosis.

## DISCUSSION

ATO is a potent anticancer agent that is highly effective against APL. However, many patients are prevented from receiving ATO due to its cardiotoxicity [24]. In this study, we detected real-time changes in sarcomere shortening and cytoplasmic Ca^2+^ transients in ARVMs. In agreement with our previous findings [16], ATO treatment impaired cell contractility and Ca^2+^ homeostasis. However, Sal B effectively attenuated or improved ATO-induced cardiac contractile and intracellular Ca^2+^ dysfunctions. Sal B treatment also increased SERCA activity in heart tissue and ARVMs. Our results also implicated ER stress-associated apoptosis inhibition in the Sal B cardioprotective mechanism. Combined with our previous report [19, 20], our findings demonstrated that Sal B protects against ATO-induced cardiotoxicity not only through its anti-oxidative and anti-apoptotic capacities, but also by modulating calcium homeostasis and suppressing ER stress.

Ca^2+^ is a crucial secondary messenger in ventricular myocytes [25]. The [Ca^2+^]_i_ transients decay rate is commonly used to characterize the speed of intracellular Ca^2+^ clearing in the cytoplasm [26]. SERCA is responsible for calcium uptake from the cytosol into the ER lumen after contraction. We found that Sal B treatment reversed the ATO-induced decline in [Ca^2+^]_i_ transients decay rate, indicating that Sal B likely enhanced SERCA activity to maintain calcium homeostasis. SERCA is controlled by a closely associated SR membrane protein, phospholamban (PLB), which has two phosphorylation sites, Ser16 and Thr17 [27]. Dephosphorylated PLB can restrain SERCA activity, and PLB phosphorylation eliminates this inhibition. In rat ventricular myocytes, SERCA and NCX are responsible for >90% and ∼7% of Ca^2+^ removal, respectively [28]. Calcium/calmodulin-dependent protein kinase II (CaMK II) regulates intracellular Ca^2+^ transport and modulates myocardial cell contractile and electrical activities [29]. A recent study associated CaMKII with arrhythmia occurrence. Increased CaMKII activity could lead to Ca^2+^ overload in cells and trigger malignant ventricular arrhythmia [30, 31]. We found that Sal B upregulated ATO-decreased SERCA and p-PLB levels, and simultaneously downregulated ATO-increased p-CaMK II and NCX levels in heart tissue and ARVMs. Therefore, Sal B ameliorated ATO-induced Ca^2+^ overload in ARVMs by regulating SERCA expression and activity.

ATO treatment can induce ATP depletion, oxidative stress, and calcium imbalance, leading to accumulation and aggregation of unfolded proteins in the ER lumen, and ultimately the unfolded protein response (UPR) and ER stress [32, 33]. Glucose-regulated proteins 78 (GRP78) are ER chaperones and serve as markers of ER stress [34]. UPR is characterized by the actions of three stress sensors: PERK, ATF6, and IRE1 [35-37]. When ER stress is not mitigated and homeostasis is not restored, the endogenous ER stress response transitions from promoting cell survival to apoptosis through CHOP-, c-Jun NH2-terminal kinase (JNK)-, and caspase-12-dependent pathways [38-41].

Although the significance of ER stress in the ATO anticancer effect has been studied, few studies focused on alleviating ATO cardiotoxicity by suppressing ER stress, and whether ATO-induces ER stress in the heart requires further study [13]. Our data showed that ATO enhanced expression of the ER stress-responsive marker, GRP78, the ER stress sensors, p-ATF6, p-PERK, eIf2α, and IRE1, and the downstream apoptosis proteins, CHOP and caspase-12. This indicated that ATO activated ER stress and, therefore, apoptosis. However, Sal B suppressed these ATO-induced processes in heart tissue and ARVMs. GRP78, p-PERK, ATF6, IRE1, CHOP, and caspase-12 were all downregulated in Sal B-treated versus ATO-treated cells. Thus, Sal B directly inhibited ATO-induced ER stress.

In conclusion, Sal B treatment protected against ATO-induced cardiotoxicity by improving contractile recovery and maintaining calcium homeostasis. We found that Ca^2+^-handling protein regulation and ER stress-associated apoptosis inhibition are important in Sal B cardioprotection *in vivo* and *in vitro*. Sal B treatment blocked Ca^2+^ overload and upregulated SERCA activity and expression, ameliorating ATO-induced calcium homeostasis imbalance. Finally, Sal B inhibited ATO-induced ER stress by downregulating GRP78, ERS, PERK, ATF6, and IRE1. Our results suggest that Sal B may prevent ATO cardiotoxicity during clinical application. However, our data do not fully explain the direct relationship between calcium homeostasis imbalance and ER stress. Therefore, the exact mechanisms of Sal B cardioprotective activity require further study.

## MATERIALS AND METHODS

### Materials

Sal B (≥98%) was purchased from Shanghai Winherb Medical S&T Development Co., Ltd. (Shanghai, China). ATO was acquired from Harbin YI-DA Pharmaceutical Ltd. (Harbin, China). Collagenase Type II and Fura-2/AM were purchased from Life Technologies Corporation (Carlsbad, CA, USA). The sarcoplasmic reticulum Ca^2+^-ATPase (SERCA) activity detection kit was acquired from Nanjing Jiancheng Bioengineering Institute (Nanjing, China). All antibodies were obtained from Santa Cruz Biotechnology except for SERCA and NCX, which were purchased from Abcam (Cambridge, UK). All chemicals were obtained from Sigma Chemical Co., Ltd. (St. Louis, MO, USA).

### Animals and treatments

Sixty male BALB/c mice weighing 18–20 g each (Vital River Laboratories, Beijing, China) were used in the experiment. All procedures were approved by the local animal committee. All animal care procedures and interventions were performed in accordance with the Guidelines and Policies for Animal Surgery provided by the Chinese Academy of Medical Sciences and Peking Union Medical College, Beijing, China, and were approved by the Institutional Animal Use and Care Committee (registration number: #IMPLAD2015090216). Experimental protocols were previously described [20]. In brief, rats were randomly divided into four groups (n=15/group): (I) the control group (10 mL/kg saline), (II) Sal B-treated group (2 mg/kg Sal B), (III) ATO-treated group (1 mg/kg ATO), and (IV) ATO + Sal B group (2 mg/kg Sal B 1 h before ATO administration). All treatments were injected once a day via the tail vein for 2 weeks. The heart was excised, and myocardial homogenates were prepared for western blotting and SERCA detection.

### Adult rat ventricular myocyte isolation

ARVMs were isolated using a modified enzymatic method as previously described [42]. Briefly, hearts were immediately excised from adult male Sprague-Dawley rats (180–200 g each) under pentobarbital sodium anesthesia (140 mg/kg intraperitoneally). Hearts were mounted on a Langendorff perfusion apparatus and perfused with Ca^2+^-containing Tyrode’s solution equilibrated with O_2_ (NaCl, 137; KCl, 5.4; MgCl_2_, 1.2; HEPES, 10; glucose, 10; and CaCl_2_, 1.2 [in mM]) for 2 min at a rate of 6 mL/min. Hearts were then perfused with Ca^2+^-free Tyrode’s solution for 5 min along with the components mentioned above, except for CaCl_2_. Hearts were then perfused with Ca^2+^-free Tyrode’s solution with collagenase Type II (210.00 units/mg) and 0.7 g/l bovine serum albumin until they became flaccid. The digested tissues were separated and filtered through a nylon mesh (300 mm) after perfusion. The filtered cell suspension was rinsed several times with Ca^2+^-containing Tyrode’s solution to progressively increase extracellular Ca^2+^ to 1.2 mM. Only viabilities over 85% and rod-shaped ARVMs with clear edges were used in this study.

### Treatment and measurement of sarcomere shortening and Ca^2+^ transients in ARVMs

Sarcomere shortening and Ca^2+^ transients were assessed simultaneously upon field stimulation (0.5 Hz with 2 ms duration, 16 V) using a video-based sarcomere contractility and calcium-recording module in a SoftEdge MyoCam system (IonOptix Corporation, Milton, MA, USA). To measure intracellular Ca^2+^, freshly isolated myocytes were incubated with Fura-2/AM (2 μM) for 15 min at 37°C and then washed twice with Ca^2+^-containing Tyrode’s solution to remove residual Fura-2/AM. ARVMs were then placed in a Warner chamber mounted on the stage of an inverted microscope (Olympus, IX-70) and superfused with the following: (I) Ca^2+^-containing Tyrode’s solution for 27 min (control group), (II) Ca^2+^-containing Tyrode’s solution with Sal B (1 μM) for 7 min (Sal B group), (III) Ca^2+^-containing Tyrode’s solution with ATO (100 μM) for 20 min (ATO group), and (IV) Ca^2+^-containing Tyrode’s solution with ATO (100 μM) for 20 min before Ca^2+^-containing Tyrode’s solution with Sal B (1 μM) for 7min (ATO + Sal B group) at a rate of 1.5 mL/min. Myocytes (*n*=30–40 cells from 5 rats per group) were allowed to equilibrate for about 10 min before measurement. Data were recorded and analyzed using IonWizard software (version 6.2.0.59) [43].

### Measurement of SERCA activity

As previously described [44], SERCA activity measurements were completed using a commercially available kit (Nanjing Jiancheng Bioengineering Institute, Nanjing, China).

### Western blot analysis

After exposure to different treatment solutions, ARVM and heart tissue homogenates were lysed on ice with tissue or cell protein extraction reagent containing 1% phenylmethylsulfonyl fluoride (CoWin Bioscience Co., Ltd., Beijing, China). Supernatants were collected after centrifuging at 12,000 rpm at 4°C for 20 min. Protein concentrations were determined using a BCA kit (Pierce Corporation, Rockford, USA). Equal amounts of protein were separated via 8–12% SDS-PAGE and then transferred to nitrocellulose membranes (Millipore Corporation, USA) in a Tris-glycine buffer at 100 V for 1 h in an icebox. Membranes were blocked with 5% (w/v) non-fat milk powder in Tris-buffered saline containing 0.1% (v/v) Tween-20 (TBST) for 3 h at room temperature, and then incubated overnight with appropriate primary antibodies (1:200) at 4°C. Membranes were washed with TBST three times, incubated with corresponding secondary HRP-conjugated antibodies (1:1000) for 2 h on a shaking table, and then washed again three times with TBST. Membranes were developed using an enhanced chemiluminescence solution. Protein levels were visualized with Image Lab Software (Bio-Rad, USA). Western blot analysis was performed as previously described [45].

### Statistical analysis

Data were expressed as means ± SD from at least three independent experiments. Statistical comparisons between different groups were determined by one-way ANOVA or the Student-Newman-Keuls method with GraphPad Prism 5.0 software (SPAA Inc., Chicago). P<0.05 was considered statistically significant.
